# Prevalence and predictors of depression among emergency physicians: a national cross-sectional study

**DOI:** 10.1186/s12888-022-03687-8

**Published:** 2022-01-28

**Authors:** Yueming Chen, Xin Shen, Jing Feng, Zihui Lei, Weixin Zhang, Xingyue Song, Chuanzhu Lv

**Affiliations:** 1 Central People’s Hospital of Zhanjiang, Zhanjiang, Guangdong China; 2 Department of Social Medicine and Health Management, School of Public Health, Tongji Medical College, Huazhong University of Science and Technology, Wuhan, Hubei China; 3 School of Public Health, Jilin University, Changchun, Jilin China; 4 Department of Emergency, Hainan Clinical Research Center for Acute and Critical Diseases, The Second Affiliated Hospital of Hainan Medical University, No. 368 Yehai Avenue, Longhua Zone, Haikou, 571199 Hainan China; 5 Emergency Medicine Center, Sichuan Provincial People’s Hospital, University of Electronic Science and Technology of China, No. 32 Yi Huan Lu Xi Er Duan, Chengdu, 610072 Sichuan Province China; 6 Research Unit of Island Emergency Medicine, Chinese Academy of Medical Sciences (No. 2019RU013), Hainan Medical University, Haikou, Hainan China

## Abstract

**Background:**

Physicians' depression can damage their physical and mental health and can also lead to prescribing errors and reduced quality of health care. Emergency physicians are a potentially high-risk community, but there have been no large-sample studies on the prevalence and predictors of depression among this population.

**Methods:**

A nationally representative cross-sectional survey of 15,243 emergency physicians was conducted in 31 provinces across China between July and September 2019. Multivariable logistic regression analysis was performed to identify predictors of depression.

**Results:**

A total of 35.59% of emergency physicians suffered from depression. Emergency physicians who were male (OR=0.91) and older [>37 and ≤43 (OR=0.83) or >43 (OR=0.71)], had high (OR=0.63) or middle (OR=0.70) level income, and participated in physical inactivity (OR=0.85) were not more likely to suffer depression. Meanwhile, those who were unmarried (OR=1.13) and smokers (OR=1.12) had higher education levels [Bachelor’s degree (OR=1.57) or Master’s degree or higher (OR=1.82)], long work tenure [>6 and ≤11 (OR=1.15) or >11;11 (OR=1.19)], poorer health status [fair (OR=1.67) or poor (OR=3.79)] and sleep quality [fair (OR=2.23) or poor (OR=4.94)], a history of hypertension (OR=1.13) and coronary heart disease (OR=1.57) and experienced shift work (OR=1.91) and violence (OR=4.94)].

**Conclusion:**

Nearly one third of emergency physicians in China suffered from depression. Targeted measures should be taken to reduce the prevalence of depression to avoid a decline in health care quality and adversely impact the supply of emergency medical services.

## Background

Depression is a chronic disease characterized by long-term low mood and lack of energy [1], which can harm mental and physical health [2, 3] and also contribute to a burden of disease [4]. Approximately 350 million people worldwide suffer from depression [5], while health care workers are the most likely to have a high rate of this disease [6]. Previous studies have shown that the prevalence rate of depression among physicians is as high as 28.8%, and 8% have admitted to suicidal ideation [7]. Physicians' depression can lead to chronic diseases [8, 9] and serious psychological problems [10] and can also adversely affect the quality of health care [11], such as increasing the incidence of medical errors and prescribing errors [12, 13].

Some potential predictors have been identified to contribute to depression among physicians, including excessive work stress and long working hours [14]. However, according to previous research, far more than general physicians (38%), emergency physicians tend to experience higher levels of working stress (more than 60%) [15, 16]. Emergency physicians often need to deal with numerous patients and various diseases while providing medical services, and they also face staffing shortages and a chaotic work environment characterized by unpredictability in the medical setting [17]. Chronic work and psychological stress have become hidden dangers to the occurrence of depression among emergency physicians.

Although the consensus is that physicians are more likely to suffer from depression than the general public [18–21], most previous studies have focused on depression among surgeons [22], primary health care workers [23], and medical students [24], with few studies focusing on emergency physicians. In reality, a growing number of studies have pointed out that the prevalence of depression varies significantly between specialties [21, 25, 26] and that targeted studies are needed according to the physician community. Previous studies have analysed the prevalence of depression among emergency physicians in the United Kingdom (N=371) [27], the United States (*N*=763) [28], and Turkey (N=192) [29], but there is still a lack of representative large-sample studies. Therefore, the purpose of this study was to explore the prevalence and influencing factors of depression among emergency physicians based on a large nationwide sample. The results of this study could provide global health care policy-makers and researchers with more accurate reports on the prevalence of depression, which can be a reference for targeted measures to safeguard the psychological health of emergency physicians and ensure the quality of emergency medical services.

## Methods

### Ethics statement

The study protocol was approved by the Institutional Ethics Board of the Second Affiliated Hospital of Hainan Medical University, Haikou, China. All individuals provided written informed consent.

### Study participants and survey design

Our study follows Strengthening The Reporting of OBservational Studies in Epidemiology

(STROBE) [30]. A cross-sectional study was carried out in China from July 2019 to September 2019. A multistage stratified random sampling design was used in this study. First, 31 Chinese provinces were classified as developed, developing, or less-developed regions according to per capital household income in 2018. Second, we randomly selected 10 hospitals from each province. Third, based on the number and scale of the hospitals, 40% of the emergency physicians who had practised in the emergency department for at least 6 months were randomly selected from each hospital to complete a self-administered questionnaire. The participants included only emergency physicians and not nurses or other technicians who provided emergency care. In our study, a 95% confidence level and ±5% precision were assumed for the following equation:

$$\mathrm{n}=\frac{N}{1+N{(e)}^2}$$

where n is the sample size, N is the population size, and e is the level of precision. Thus, the conservative total sample size for this questionnaire was 1400.

### Instrument and measurement

The questionnaire was designed based on literature reviews, group discussions, and preliminary interviews. Furthermore, a pilot study was conducted in one Wuhan community to improve the quality of the questionnaire. This questionnaire focuses on the physical and mental health and professional attitude of emergency physicians, and the relevant parts of this study include sociodemographic information (e.g., region, age, gender, education level, marital status, and professional title), workplace violence and depression.

#### The Chinese version of the Workplace Violence Scale (WVS)

The WVS was developed by Wang et al. [31] and has good reliability and validity for measuring the incidence of workplace violence when applied to medical staff in China. It includes 5 items measured with a four-point ordinal scale ranging from 0 (never) to 3 (more than 3 times/year). In this study, the Cronbach’s alpha for WPV was 0.81.

#### The Center for Epidemiological Studies Depression scale (CES-D)

The CES-D was used to assess depressive symptoms. The scale includes 20 items; each item is scored on a four-point scale ranging from 0 ("little or none of the time") to 3 ("most or almost all of the time"). The total score ranges from 0 to 60 points, and the higher the score, the more severe the depressive symptoms. On the original CES-D scale, a total score of 16 was used to detect the presence of depressive symptoms [32]. However, many studies have assessed the diagnostic accuracy of CES-D in detecting depression in the general population and have proposed multiple cut-off points, such as a cut-off point of 18 for elderly people living in residential homes [33] and a cut-off score of 22 for older Chinese individuals [34]. A meta-analysis systematically reviewed 28 CES-D studies, including several Chinese studies, and obtained an optimal cut-off of 20 points [35]. As a result, an overall score of 20 or higher was considered an indicator of depressive symptoms in this study, consistent with previous research [36]. The CES-D has good reliability and validity, and it has been widely used in the Chinese population. In this study, the Cronbach's alpha coefficient of the scale was 0.90.

### Data collection and quality control

A web link to the online questionnaire, which was designed using Questionnaire Star, was disseminated to the participants through WeChat (similar to WhatsApp in Western countries, WeChat is the largest communication platform in China, with over one billion users). To prevent the same participants from repeatedly answering the questionnaire, each device (e.g., smartphone or computer) was eligible to complete the questionnaire only once, and logical checks were concurrently run on the WeChat platform to identify invalid questionnaires. The data were entered into a web-based database by trained investigators to ensure accuracy.

### Statistical methods

Descriptive analyses used means for the continuous variables and percentages for the categorical data. The descriptive statistics for the categorical variables were reported as the frequency (percentage) and were compared between having depression (CES-D Scores ≥20) or not using Pearson’s *χ*^*2*^ test. Depression was treated as the dependent variable and was a categorical variable. Multivariable logistic analysis (cut-offs for selection and elimination: *P*=0.05 and *P*=0.10, respectively) was used to calculate the odds ratios (ORs) and 95% confidence intervals (CIs) for predictors that might be associated with the prevalence of depression. All analyses were performed using STATA 13.0, and all tests were two sided with a significance level of 0.05.

## Results

In total, 15,455 emergency physicians were asked to participate in this survey, and 182 physicians did not respond. Additionally, 30 questionnaires were discarded due to missing information. Ultimately, 15,243 eligible questionnaires were used in this analysis. Table 1 presents the main characteristics of the survey respondents. Among the 15,243 respondents, most were married (83.26%), were men (69.87%) and had a Bachelor’s degree or above (94.18%). The mean age (SD) was 37.66 (8.06) years. Approximately 65% of the participants had intermediate or senior professional titles. In total, 55.23% of the respondents were in the middle-income and lower-income groups, more than half estimated their own health to be at the general level, and approximately 40% were from developing regions. A minority of the participants (27.19%) participated in regular physical activity. Overall, 89.87% of respondents reported exposure to WPV in the previous 12 months (Table 1).

**Table 1:** Statistical description of study samples

Variables	N (%)	Depression (CES-D Scores ≥20)	*χ*^*2*^	*P* value
Total	15243 (100.00)	5425 (35.59)	NA	NA
Gender				
Male	10650 (69.87)	3854 (36.19)	5.50	**0.02**
Female	4593 (30.13)	1571 (34.20)		
Age group, y				
≤31	4089 (26.83)	1467 (35.88)	146.60	**<0.01**
>31 and ≤37	4117 (27.01)	1674 (40.66)		
>37 and ≤43	3291 (21.59)	1236 (37.56)		
>43	3746 (24.57)	1048 (27.98)		
Region				
Developed	6000 (39.36)	2031 (33.85)	13.13	**0.01**
Middle-developed	4097 (26.88)	1499 (36.59)		
Less-developed	5146 (33.76)	1895 (36.82)		
Education level				
Associate’s degree or vocational diploma	894 (5.86)	230 (25.73)	41.21	**<0.01**
Bachelor degree	10293 (67.53)	3702 (35.97)		
Master degree or higher	4056 (26.61)	1493 (36.81)		
Marital status				
Married/widow/divorced	12691 (83.26)	4494 (35.41)	1.06	0.30
Unmarried	2552 (16.74)	931 (36.48)		
Income status				
High	1672 (10.97)	398 (23.80)	312.27	**<0.01**
Middle	6746 (44.26)	2106 (31.22)		
Low	6825 (44.77)	2921 (42.80)		
Work tenure, y				
≤3	4921 (32.28)	1660 (33.73)	48.49	**<0.01**
>3 and ≤6	3114 (20.44)	1173 (37.67)		
>6 and ≤11	3424 (22.46)	1351 (39.46)		
>11	3784 (24.82)	1241 (32.80)		
Contract status				
Permanent	9715 (63.73)	3430 (35.31)	0.94	0.33
Temporary	5528 (36.27)	1995 (36.09)		
Professional title				
Elementary or below	5349 (35.09)	1933 (36.14)	103.13	**<0.01**
Intermediate	5861 (38.45)	2305 (39.33)		
Senior	4033 (26.46)	1187 (29.43)		
Ownership				
Governmental	14599 (95.78)	5246 (35.93)	17.82	**<0.01**
Non-governmental	644 (4.22)	179 (27.80)		
Level of hospital				
Three-grade level	10152 (66.60)	3661 (36.06)	20.16	**<0.01**
Two-grade level	4841 (31.76)	1708 (35.28)		
Other	250 (1.64)	56 (22.40)		
Shift work				
Yes	13288 (87.17)	5031 (37.86)	233.12	**<0.01**
No	1955 (12.83)	394 (20.15)		
Workplace violence				
Yes	13699 (89.87)	5267 (38.45)	481.88	**<0.01**
No	1544 (10.13)	158 (10.23)		
Self-perceived health status				
Good	4707 (30.88)	811 (17.23)	1781.32	**<0.01**
Fair	7729 (50.71)	2778 (35.94)		
Poor	2807 (18.41)	1836 (65.41)		
BMI (kg/m^2^)				
<25	9588 (62.90)	3345 (34.89)	5.57	**0.02**
≥25	5655 (37.10)	2080 (36.78)		
History of hypertension				
Yes	2317 (15.20)	1000 (43.16)	68.29	**<0.01**
No	12926 (84.80)	4425 (34.23)		
History of diabetes				
Yes	598 (3.92)	257 (42.98)	14.81	**<0.01**
No	14645 (96.08)	5168 (35.29)		
History of coronary heart disease				
Yes	471 (3.09)	273 (57.96)	106.11	**<0.01**
No	14772 (96.91)	5152 (34.88)		
Smoking status				
Nonsmokers	12287 (80.61)	4265 (34.71)	21.34	**<0.01**
Smokers	2956 (19.39)	1160 (39.24)		
Alcohol drinking				
Nondrinkers	11451 (75.12)	4068 (35.53)	0.08	0.77
Drinkers	3792 (24.88)	1357 (35.79)		
Physical inactivity				
Yes	4145 (27.19)	1113 (26.85)	189.64	**<0.01**
No	11098 (72.81)	4312 (38.85)		
Sleep quality				
Good	2295 (15.06)	250 (10.89)	1777.99	**<0.01**
Fair	7347 (48.20)	2058 (28.01)		
Poor	5601 (36.74)	3117 (55.65)		

Overall, 5425 participants acquired CES-D scores equal to or greater than 20, which indicated that 35.59% of emergency physicians could suffer from depression. The factors associated with this psychological problems are presented in Table 2. Emergency physicians who were male (OR=0.91) and older [>37 and ≤43 (OR=0.83) or >43 (OR=0.71)], had high (OR=0.63) or middle (OR=0.70) level income, and participated in physical inactivity (OR=0.85) were not more likely to suffer depression. Meanwhile, those who were unmarried (OR=1.13) and smokers (OR=1.12) had higher education levels [Bachelor’s degree (OR=1.57) or Master’s degree or higher (OR=1.82)], long work tenure [>6 and ≤11 (OR=1.15) or >11;11 (OR=1.19)], poorer health status [fair (OR=1.67) or poor (OR=3.79)] and sleep quality [fair (OR=2.23) or poor (OR=4.94)], had a history of hypertension (OR=1.13) and coronary heart disease (OR=1.57), and experienced violence (OR=1.91) and violence (OR=4.94)].

**Table 2:** Logistic Analysis on the Influencing Factors of Emergency physicians’ depression in China

Variables	Coefficient	*S.E.*	*P*	*OR*	95% *CI*
Gender (Ref: Female)					
Male	-0.10	0.05	**0.04**	0.91	0.83 – 0.10
Age group, y (Ref: ≤31)					
>31 and ≤37	-0.09	0.07	0.16	0.91	0.80 – 1.04
>37 and ≤43	-0.19	0.08	**0.02**	0.83	0.71 – 0.97
>43	-0.34	0.10	**<0.01**	0.71	0.59 – 0.86
Region (Ref: Less-developed)					
Developed	-0.07	0.05	0.12	0.93	0.85 – 1.02
Middle-developed	-0.02	0.05	0.73	0.98	0.89 – 1.08
Education level (Ref: Associate’s degree or vocational diploma)					
Bachelor degree	0.45	0.09	**<0.01**	1.57	1.31 – 1.88
Master degree or higher	0.60	0.10	**<0.01**	1.82	1.49 – 2.23
Marital status (Ref: Married/widow/divorced)					
Unmarried	0.12	0.06	**0.04**	1.13	1.01 – 1.26
Income level (Ref: Low)					
High	-0.46	0.07	**<0.01**	0.63	0.55 – 0.73
Middle	-0.35	0.04	**<0.01**	0.70	0.65 – 0.76
Work tenure, y (Ref: ≤3)					
>3 and ≤6	0.01	0.06	0.87	1.01	0.90 – 1.13
>6 and ≤11	0.14	0.06	**0.02**	1.15	1.02 – 1.30
>11	0.17	0.07	**0.01**	1.19	1.04 – 1.36
Contract status (Ref: Temporary)					
Permanent	0.07	0.04	0.11	1.07	0.98 – 1.17
Professional title (Ref: Elementary or below)					
Intermediate	0.06	0.06	0.36	1.06	0.94 – 1.19
Senior	0.06	0.08	0.48	1.06	0.90 – 1.25
Ownership (Ref: Non-governmental)					
Governmental	0.18	0.10	0.07	1.20	0.98 – 1.46
Level of hospital (Ref: Other)					
Three-grade level	0.34	0.17	0.04	1.41	1.01 – 1.97
Two-grade level	0.33	0.17	0.05	1.40	1.00 – 1.95
Shift work (Ref: No)					
Yes	0.32	0.07	**<0.01**	1.38	1.20 – 1.58
Workplace violence (Ref: No)					
Yes	1.26	0.09	**<0.01**	3.51	2.94 – 4.20
Self-perceived health status (Ref: Good)					
Fair	0.51	0.05	**<0.01**	1.67	1.52 – 1.84
Poor	1.33	0.06	**<0.01**	3.79	3.35 – 4.29
BMI (kg/m^2^)(Ref: <25)					
≥25	0.01	0.04	0.85	1.01	0.93 – 1.09
History of hypertension (Ref: No)					
Yes	0.12	0.06	**0.03**	1.13	1.01 – 1.26
History of diabetes (Ref: No)					
Yes	0.12	0.10	0.23	1.13	0.93 – 1.38
History of coronary heart disease (Ref: No)					
Yes	0.45	0.11	**<0.01**	1.57	1.26 – 1.95
Smoking status (Ref: Nonsmokers)					
Smokers	0.11	0.05	**0.03**	1.12	1.01 – 1.24
Alcohol drinking (Ref: Nondrinkers)					
Drinkers	0.04	0.05	0.37	1.05	0.95 – 1.15
Physical inactivity (Ref: No)					
Yes	-0.16	0.05	**0.01**	0.85	0.78 – 0.94
Sleep quality (Ref: Good)					
Fair	0.80	0.08	**<0.01**	2.23	1.92 – 2.58
Poor	1.60	0.08	**<0.01**	4.94	4.24 – 5.75

## Discussion

To our knowledge, this is the largest cross-sectional survey of physicians' depression in the world, and we focused on emergency physicians, a community of physicians with high levels of work stress. We found that more than one-third of emergency physicians (35.59%) suffered from depression. Compared to previous studies of surgeons (20%) [22], primary health care workers (18%) [23] and medical students (28.8%) [24], emergency physicians showed higher levels of depression, which indicates that full attention should be given to this community. In addition, we identified a number of representative factors that clearly correlate with the increase in depression among emergency physicians, which could provide references for targeted measures.

### Age, gender, marital status and income level

Our study found that emergency physicians who were older, male and had higher income levels were less likely to suffer from depression. These findings are consistent with previous studies. Liang et al. [37] reported that younger medical workers had higher self-rated depression scores than older medical workers. Erdur et al. [29] found that female and lower-paid emergency physicians were more likely to be depressed. Single doctors were more likely to report psychiatric symptoms [38], which is consistent with our findings. Although financial difficulties and additional responsibilities of family life may increase the likelihood of negative psychological effects on doctors, the family's social support mechanisms appear to play a role in preventing depression and anxiety [29]. Podder et al. [39] also mentioned that unmarried physicians are more stressed and more likely to have psychological problems. These findings suggest that more attention should be given to emergency physicians who are younger, female, unmarried and have low income.

### Education level and work tenure

Well-educated workers tend to have higher career goals [40]. If career opportunities within the organization are limited, they are more likely to develop psychological problems [41]. Our study found that emergency physicians with higher education levels are more likely to develop depression, which could mean that these communities need adequate improvement opportunities. In addition, sense of achievement is an important factor that affects work enthusiasm [42]. Emergency physicians with longer working tenure may experience a decreased sense of professional accomplishment. Longer working tenure may lead to fatigue, tension and other psychological problems [43]. Erdur et al. [29] reported that emergency physicians with more working years were more likely to be depressed, and those who had been working in emergency departments for more than 10 years had the highest depression scores. Based on these results, one possible suggestion is that maintaining a sense of passion for work is a potential way to prevent depression among emergency physicians.

### Health status, sleep quality, physical inactivity and smoking

Many studies have shown that a decline in health and sleep quality is an important factor in the development of depression [44–46]. Hypertension and coronary heart disease have also been reported to be associated with the occurrence of depression [47–49]. Our study also showed that emergency physicians with poor physical health and sleep quality were more likely to report depression. Emergency physicians who often participate in physical activity were less likely to be depressed, which is consistent with other studies on the relationship between exercise and depression [50, 51]. In addition, smoking can be a reflection of depression. More specifically, it can be a mechanism of coping with depression [52]. A previous study reported that emergency department physicians had higher substance use rates than physicians in other specialties [53]. Erdur et al. also mentioned that emergency department physicians who smoked were markedly more likely to report psychological problems [29], which is in accordance with our results. Therefore, the managers of health institutions should focus on the health status of emergency physicians, while the physician community should develop healthy living habits. These measures could prevent psychological problems among these physicians.

### Shift work and workplace violence

Because of scarce medical resources and a growing number of patients, emergency physicians are often required to work shifts [54]. Previous studies have shown that shift work is a risk factor for depression, anxiety and sleep disorders. Excessive shift work can lead to emergency physicians' depression. On the other hand, workplace violence in emergency departments has received increasing attention in recent years [55, 56]. A survey of the Occupational Health Safety Network (OHSN) in America during 2012–2015 showed that the incidence of emergency room violence was 19.3%, with an average annual growth rate of 23% [57], and previous studies have shown that only 30% of cases were documented [58, 59], while nearly 80% of cases were undocumented [60]. Wang et al. [61] found that workplace violence is a contributing factor to depression among physicians and that reducing violence would be beneficial to promoting the mental health status of medical staff, which is consistent with our findings. Therefore, targeted measures are needed to optimize the health service system, provide more flexible working regimes for emergency physicians, and mitigate doctor-patient relationships to reduce workplace violence.

China has the largest number of emergency physicians in the world, with nearly 60,000, accounting for more than 2.1% of the total physicians, and it also receives the largest number of emergency patients each year, at more than 166.5 million [62]. This study found that 35.59% of them suffered from depression, which is higher than previous findings in the United Kingdom (18%) [27], the United States (19.5%) [28] and Turkey (29%) [29], suggesting that there are different depression prevalences in different regions. Although emergency physicians in many countries also face intense work pressure and excessive numbers of patients, such as in China, the results of this study should still be interpreted with caution, and further exploration should be undertaken.

### Strengths, limitations and future suggestions

Our study used a large-sample cross-sectional survey to explore the influencing factors of depression among emergency physicians. First, the large sample size significantly increased the statistical power. We surveyed nearly a quarter of the nation's emergency physicians; thus, the participants were representative. Second, this study included more than twenty potential contributors and produced many valuable findings, which reflects their working status and presents a broad view of the challenges faced by emergency physicians. Third, the survey was anonymous and self-administered, which likely made the respondents provide more valid responses and eliminated interviewer bias.

This study had several limitations. First, the study used a cross-sectional study design, which precluded the evaluation of the temporality of the observed relationships. Second, the data were collected from the participants’ self-reports; thus, recall bias was unavoidable. Finally, the study was performed before the COVID-19 pandemic, which may have led to changes in the depression of emergency physicians after the pandemic.

Based on our findings, we suggest that, first, prospective studies are needed to investigate the association between the identified factors and depression. The identification of depression at a particular point in time or over a period of time is important, and further exploration is needed. Second, this study highlights the need for the investigation or implementation of interventions to improve emergency physicians’ mental health or promote strategies to reduce work pressure in health care settings. Finally, investigating the potential impact of depression on emergency physicians’ work performance, the quality of patient care delivery, and family life would provide important insights.

## Conclusion

Our study found that nearly one third of emergency physicians in China suffered from depression, which is influenced by a variety of factors. Targeted measures should be taken to reduce the prevalence of depression to avoid a decline in health care quality and adversely impact the supply of emergency medical services. Emergency physicians who are female; younger; unmarried; smokers; have a low salary, higher education level, long working tenure, poor health status and sleep quality, history of hypertension and coronary heart disease; are frequent participants; and experience shift work and workplace violence should be given more attention.

## Data Availability

Data may be made available by contacting the corresponding author.

## Abbreviations

BMI: Body Mass Index
CES-D: Center for Epidemiological Studies-Depression Scale
